# *Leishmania braziliensis* replication protein A subunit 1: molecular modelling, protein expression and analysis of its affinity for both DNA and RNA

**DOI:** 10.1186/s13071-014-0573-8

**Published:** 2014-12-12

**Authors:** Paola A Nocua, Cesar A Ramirez, George E Barreto, Janneth González, José M Requena, Concepción J Puerta

**Affiliations:** Laboratorio de Parasitología Molecular, Facultad de Ciencias, Pontificia Universidad Javeriana, Carrera 7 No 43-82, Edificio 50, Laboratorio 113, Bogotá, Colombia; Departamento de Nutrición y Bioquímica, Facultad de Ciencias, Pontificia Universidad Javeriana, Bogotá, Colombia; Centro de Biología Molecular Severo Ochoa (CSIC-UAM), Universidad Autónoma de Madrid, Madrid, Spain

**Keywords:** Replication protein A (RPA), RPA subunit 1, Single-stranded DNA binding protein, RNA binding protein, *Leishmania braziliensis*

## Abstract

**Background:**

Replication factor A (RPA) is a single-strand DNA binding protein involved in DNA replication, recombination and repair processes. It is composed by the subunits RPA-1, RPA-2 and RPA-3; the major DNA-binding activity resides in the subunit 1 of the heterotrimeric RPA complex. In yeast and higher eukaryotes, besides the three basic structural DNA-binding domains, the RPA-1 subunit contains an N-terminal region involved in protein-protein interactions with a fourth DNA-binding domain. Remarkably, the N-terminal extension is absent in the RPA-1 of the pathogenic protozoan *Leishmania (Leishmania) amazonensis*; however, the protein maintains its ability to bind ssDNA. In a recent work, we identify *Leishmania (Viannia) braziliensis* RPA-1 by its specific binding to the untranslated regions of the *HSP70* mRNAs, suggesting that this protein might be also an RNA-binding protein.

**Methods:**

Both rLbRPA-1 purified by His-tag affinity chromatography as well as the *in vitro* transcribed *L. braziliensis* 3′ HSP70-II UTR were used to perform pull down assays to asses nucleic acid binding properties. Also, homology modeling was carried out to construct the LbRPA-1 tridimensional structure to search relevant amino acid residues to bind nucleic acids.

**Results:**

In this work, after obtaining the recombinant *L. braziliensis* RPA-1 protein under native conditions, competitive and non-competitive pull-down assays confirmed the single-stranded DNA binding activity of this protein and demonstrated its interaction with the 3′ UTR from the *HSP70-II* mRNA. As expected, this protein exhibits a high affinity for ssDNA, but we have found that RPA-1 interacts also with RNA. Additionally, we carried out a structural analysis of *L. braziliensis* RPA-1 protein using the X-ray diffraction structure of *Ustilago maydis* homologous protein as a template. Our results indicate that, in spite of the evolutionary divergence between both organisms, the structure of these two RPA-1 proteins seems to be highly conserved.

**Conclusion:**

The LbRPA-1 protein is a ssDNA binding protein, but also it shows affinity *in vitro* for the *HSP70* mRNA; this finding supports a possible *in vivo* role in the *HSP70* mRNA metabolism. On the other hand, the three dimensional model of *Leishmania* RPA-1 serves as a starting point for both functional analysis and its exploration as a chemotherapeutic target to combat leishmaniasis.

## Background

The leishmaniasis, caused by protozoan parasites belonging to the genus *Leishmania,* affects the population of 98 countries on the 5 continents, being 350 million people at risk of infection, 12 million are currently infected and each year 2 million new cases occur [1]. In the Americas, *Leishmania (Viannia) braziliensis* is the major agent of mucocutaneous leishmaniasis (LMC) and one of the six causative species of cutaneous leishmaniasis (LC) [2]. As there are no vaccines against any type of leishmaniasis and the treatment options are limited, efforts for developing effective vaccines and drugs should be done urgently.

Replication protein A (RPA) is the main eukaryote single-stranded DNA (ssDNA) binding protein, being essential for DNA replication, recombination and repair processes [3]. It has also been involved in cell cycle and DNA damage checkpoint activation [3,4]. In mammals, this protein, composed by the subunits RPA-1 (70 kDa), RPA-2 (32–34 kDa) and RPA-3 (14 kDa), plays two functional roles. On the one hand, the protein maintains ssDNA in an extended structure and protects solvent-exposed DNA bases from undesired chemical modifications. On the other hand, RPA interacts with several proteins in order to orchestrate different cellular processes regarding DNA maintenance [5].

The RPA heterotrimer consists of six ssDNA binding domains (DBD), also known as OB (oligonucleotide/oligosaccharide-binding) fold, each one consisting of five β-strands arranged in a β-barrel [6]. The major DNA-binding activity is found in the subunit 1 of the RPA protein (RPA-1), which is also responsible for interaction with replication and repair proteins. It exhibits a modular structure having four out of the six RPA DBDs existing in the heterotrimeric RPA. These domains, arranged in tandem, are denoted as DBD-F, DBD-A, DBD-B, and DBD-C [7,8]. The N-terminal region (RPA1N), besides bearing the DBD-F domain, is involved in interactions with other DNA metabolism proteins [9,10]. Indeed, the initiation of the DNA damage response by RPA is mediated by the RPA-1 subunit through protein-protein interactions involving its N-terminal domain [11].

A homologue of RPA was biochemically purified from the trypanosomatid *Crithidia fasciculata* by Brown and co-workers [12]. The purified complex was found to consist of three polypeptides of 51, 28, and 14 kDa that binds single-stranded DNA via the large subunit; the complex was localized within the nucleus. In a subsequent work, the genes encoding the 51-kilodalton subunit (p51) and the 28-kilodalton subunit (p28) of RPA were cloned and sequenced [13]. The predicted p51 polypeptide has sequence similarity to the corresponding subunits from humans and *Saccharomyces cerevisiae*, but it is smaller, lacking of a segment of approximately 20 kDa in the N-terminal region. In *Leishmania amazonensis,* a species belonging to the subgenera *Leishmania* and associated with different clinical manifestations such as cutaneous (CL), mucocutaneous (MCL) and visceral (VL) leishmaniasis [14]*,* the subunit 1 of replication protein A (RPA-1) was identified by its association with G-rich telomeric sequences [15]. Of note, this protein also showed affinity for RNA oligonucleotides containing the cognate telomeric sequence [15]. After sequencing of the gene and deducing of the encoded amino acid sequence, it was found that LaRPA-1, like the CfRPA-1 (see above), lacked the N-terminal RPA70N domain (present in human and yeast RPA-1), but shared with hRPA-1 and yRPA-1 a canonical N-terminal tRNA_anti domain, which is an OB (oligonucleotide/oligosaccharide-binding) fold structure [16]. More recently, these authors found evidence that the natural absence of RPA1N domain in LaRPA-1 does not impair its participation in DNA damage response and telomere protection [17].

In a recent work, we identified the *L. braziliensis* RPA-1 (LbRPA-1) protein based on its binding to the 5′ and 3′ untranslated regions (UTR) of the 70 kDa heat shock protein (*HSP70)* mRNAs [18]. In order to further analyze the role played by LbRPA-1, as a first step, we conducted a structural analysis and its tertiary structure was modelled based on the X-ray diffraction structure of *Ustilago maydis* RPA-1 protein, which has been solved recently [19]. In spite of the sequence divergence existing between both proteins, *L. braziliensis* RPA-1 protein conforms the typical DNA-binding domains of RPA proteins. Additionally, we expressed the LbRPA-1 protein in *Escherichia coli* and the purified recombinant protein was used to study its affinity for both DNA and RNA. As expected, the rLbRPA-1 binds ssDNA with high affinity, but remarkably it also binds RNA, suggesting a possible role for this protein in mRNA metabolism.

## Methods

### Parasite cultures and nucleic acid extraction

Promastigotes of *L. braziliensis* MHOM/BR/75/M2904 were cultured *in vitro* at 26°C in Schneider’s insect medium (Sigma Aldrich, Inc., St. Louis, MO, USA) supplemented with 20% heat-inactivated fetal calf serum (Eurobio, Inc., Les Ulis, France), and 0.1 μg/mL of 6-biopterin (Sigma Aldrich, Inc., St. Louis, MO, USA). Total DNA from parasite cells was isolated using the phenol-chloroform-isoamilic alcohol method [20].

### Cloning and sequence analysis of the *L. braziliensis* RPA-1 gene

The *L. braziliensis* RPA-1 (LbRPA-1) coding region was amplified from genomic DNA by PCR using the primers RPA1F (5′-*GGATCC*ATCGTGATGCAGCAGCCG-3′) and RPA1R (5′-*CTGCAG*TCACACGTACGCCTCGATGAG-3′). These primers were designed from the LbrM.28.1990 entry existing in the *L. braziliensis* GeneDB database (http://genedb.org). These primers amplify a fragment of 1422 bp and contain the *Bam*HI and *Pst*I restriction sites (italics letters in the sequence), respectively. The PCR mix reaction (final volume of 20 μL) included: 1× reaction buffer (10 mM Tris–HCl pH 9.0, 50 mM KCl, 0.1% Triton X-100), 2 mM MgCl_2_, 0.4 mM of dNTP mix, 0.5 μM of each primer, 0.06 units per μL of expand high fidelity enzyme (Roche, Inc., Mannheim, Germany) and 3.3 ng/μL of *L. braziliensis* total DNA. An MJ Research PTC-100 DNA thermocycler was used for the reaction with the following amplification profile: 95°C/3 min, 3 cycles at 92°C/30 s, 56°C/30 s, 72°C/1.5 min, followed by 36 cycles at 92°C/30 s, 64°C/30 s, 72°C/1.5 min, and a final extension at 72°C for 10 min and 12°C as PCR stop temperature. The amplified fragment was resolved in agarose gels and visualized under UV exposure after ethidium bromide staining. PCR product was excised from gel, purified using a Zymoclean™ Gel DNA Recovery Kit (Zymo Research, Irvine, CA, USA) and cloned into the pGEM-T Easy plasmid (Promega, Inc., Fitchburg, WI, USA). The plasmid insert of two clones, named RPA-1A and RPA-1B, was sequenced using the Big Dye Terminators v3.1 kit (Applied Biosystem, Foster City, CA, USA) by automatic sequencing at the Servicio de Genómica (Parque Científico de Madrid, Universidad Autónoma de Madrid). The sequence of both clones was identical to the entry LbrM.28.1990.

The homology analyses of amino acids sequences of RPA-1 was carried out using the T-Coffee tool (http://www.ebi.ac.uk/Tools/msa/tcoffee/) [21]. The domains and potential phosphorylation sites were identified by the InterProScan tool (http://www.ebi.ac.uk/Tools/pfa/iprscan/) [22,23] and the NetPhos server (http://cbs.dtu.dk/services/NetPhos/) [24], respectively.

### Cloning and expression of the *L. braziliensis* RPA-1 gene

The *LbRPA1* coding region was subcloned into the pQE30 expression plasmid (QIAGEN, Inc., Hilden, Germany), and thermo competent *E. coli* cells (M15 strain) were transformed with the corresponding plasmid. The cells were grown in 200 mL of LB medium supplemented with 75 μg/mL of ampicillin and 25 μg/mL of kanamycin overnight at 37°C. When the culture reached an A_600_ of 0.6, protein expression was induced with 1 mM isopropyl thio-β-galactopyranoside (IPTG); bacteria were incubated at 37°C for an additional 4 h. The cells were harvested (15 min, 4696 *g* at 4°C) and the pellet was suspended in 10 mL of lysis buffer (300 mM NaCl, 50 mM NaH_2_PO_4_, 0.5 mg/mL lysozyme, 10 μg/mL DNase I, 10 μg/mL RNase A and 1% Triton X-100, pH 8.0), incubated on ice for 30 min and then 1 mM PMSF was added. This mixture was centrifuged, and the pellet was suspended in 10 mL of Urea lysis buffer A (100 mM NaH_2_PO_4_, 10 mM Tris–HCl and 8 M Urea, pH 8.0), and disrupted by sonication on ice followed by centrifugation. The presence of the protein in the supernatant was confirmed by SDS-PAGE.

Protein purification was carried out through affinity chromatography using a Ni^2+^-NTA-Agarose resin (QIAGEN, Inc., Hilden, Germany), and following the method described by Lira and co-workers [25], with a few modifications. In brief, after binding to the nickel-column, the protein was refolded passing a urea gradient (from 6 M to 1 M) in a solution containing 50 μg/mL heparin, 300 mM NaCl, 100 mM NaH_2_PO_4_ and 20% glycerol. Subsequently the protein was eluted in different fractions with a buffer containing 50 mM NaH_2_PO_4_, 300 mM NaCl, 400 mM imidazole, 10 mM HEPES, 50 μg/ml heparin, 5% glycerol and 50 mM glycine, pH 7.5. The concentration of refolded protein was determined using the Micro BCA™ protein assay kit (Thermo Scientific, Inc., Waltham, MA, USA). Finally, the purity of the protein was checked by SDS-PAGE.

### Cloning and expression of the *L. braziliensis α-tubulin* gene

For cloning the *α-tubulin* ORF of *L. braziliensis*, two oligonucleotides were designed from the entry LbrM13_V2.0200: Lb-Tub-F, *GGATCC*ATGC GTGAGGCTAT CTGC (*Bam*HI site in italics letters); Lb-Tub-R, *CTGCAG*CTAG TACTCCTCGA CGTCCT (*Pst*I site in italics letters). PCR reaction was carried out as indicated above, using the following amplification program: 95°C for 5 min, 30 repeated cycles of 30 s at 95°C, 30 s at 58°C, and 2 min at 72°C. A final extension of 5 min at 72°C was included. The PCR product was digested with *Bam*HI and *Pst*I and cloned into the corresponding restriction sites of plasmid pQE30 (QIAGEN, Inc., Hilden, Germany). After checking the sequence correctness, the resulting clone was used for protein expression in *E. coli* M15 strain. Protein expression and purification was done following identical procedures to those used for the rLbRPA-1 protein (see above).

### *In vitro* transcription and non-radioactive labeling of the *L. braziliensis* 3′ *HSP70-II* UTR

The *L. braziliensis* 3′ *HSP70-II* UTR region was PCR amplified as described previously using pTLb3H70-11B clone as a template [18]. The T7 promoter sequence was included in the forward oligonucleotide in order to obtain a PCR product bearing at its 5′-end the recognition site for the T7 RNA polymerase. For *in vitro* transcription, the MEGAscript® T7 kit was used according to manufacturer’s instructions (Ambion, Inc., Austin, TX, USA). Subsequently, the transcription products were treated with RNase free-DNase and the RNAs purified by the TRIZOL reagent method (Invitrogen, Inc., Carlsbad, CA, USA) according to Ramirez and coworkers [18]. The non-radioactive labeling was performed using a terminal transferase (Roche, Inc., Mannheim, Germany) following the manufacturer’s instructions.

### Binding and competition assays of the *L. braziliensis* RPA-1 recombinant protein to a digoxigenin-labelled oligodeoxyribonucleotide

The rLbRPA-1 protein (40 μg) was incubated with 0.8 mg of His-tag isolation & Pull down dynabeads (Invitrogen, Inc., Carlsbad, CA, USA) in 1× binding buffer (50 mM NaH_2_PO_4_, 300 mM NaCl, 50 μg/ml heparin, 0.01% tween 20 and 5% glycerol) at room temperature for 10 min on continuous stirring. Unbound protein was removed through three rinses with 500 μl of 1× binding buffer. Then, the rLbRPA-1 bound to the beads was incubated with 12.5 ng of Dig-Oligodeoxyribonucleotide (Dig-O, 5′ Dig-GGCGAGCAGC GTCGGCCACG CATCTCAACG CGAGCGTGCT 3′) and either double stranded DNA (dsDNA), single stranded DNA (ssDNA), yeast tRNA (Roche, Inc., Mannheim, Germany) or none. Plasmid pQE30 DNA (QIAGEN, Inc., Hilden, Germany) was linearized with *Bam*HI and used as dsDNA competitor; the ssDNA competitor was obtained after incubation at 95°C for 10 min of the *Bam*HI-linearized pQE30. After incubation of the mixtures overnight at room temperature in 1× pull-down buffer (3.25 mM NaH2PO4 pH 7.4, 70 mM NaCl, and 0.01% tween 20); the protein-nucleic acid complexes loaded in the beads were crosslinked by adding 1% formaldehyde, and afterwards the beads were washed three times with 1× pull down buffer. Finally, the protein-nucleic acid complexes were eluted in 20 μL of pull down elution buffer (50 mM NaH2PO4, 300 mM NaCl, 400 mM Imidazole, 10 mM HEPES, 50 μg/mL heparin and 10% glycerol) at 90°C for 5 min. The rLbRPA1-nucleic acid complexes obtained were analyzed by dot immunoblotting. Immunological detection of the complexes was performed using the DIG luminescent detection kit (Roche, Inc., Mannheim, Germany). For this purpose, 2 μL of the eluted samples were placed on a nylon membrane (Roche, Inc., Mannheim, Germany), fixed with UV (1200 μJ), washed with maleic washing buffer (0.1 M maleic acid, 0.15 M NaCl pH 7.5 and 0.3% tween 20), and blocked overnight on continuous stirring. Next, the membrane was incubated with anti-DIG-AP conjugate (1:20000 in blocking solution) at 37°C during 40 min on continuous stirring. After extensive washing with the maleic washing buffer, the membrane was incubated in the detection buffer (0.1 M Tris–HCl and 0.1 M NaCl pH 9.5); afterwards, the CSPD substrate solution was spread on membrane, which was incubated for 10 min at 37°C. Finally, an x-ray film (Agfa, Inc., Mortsel, Belgium) was exposed to the membrane for 30 min and then developed.

### Pull down assays of the *L. braziliensis* RPA-1 recombinant protein and nucleic acids

The rLbRPA-1 protein was incubated with His-tag isolation & Pull down dynabeads (Invitrogen, Inc., Carlsbad, CA, USA) in binding 1× buffer at room temperature for 15 min on continuous stirring. Unbound protein was removed and the protein-coupled beads were blocked by adding 5 μg of recombinant *L. braziliensis* α-tubulin for 20 min. Then, the beads were incubated with single strand DNA or RNA for 20 min in pull-down 1× buffer; afterwards, the beads were washed three times with pull down buffer 0.5× and once with DEPC-treated distilled water. Finally, the protein-nucleic acid complexes were eluted in 10 μL of pull down elution buffer at 90°C for 5 min. The rLbRPA1-nucleic acid complexes obtained were analyzed by dot immunoblotting and the signals were detected as mentioned above.

### Molecular modeling of the Leishmania RPA-1 proteins

The primary sequences were analyzed by PSI-BLAST (Position-Specific Iterated-Basic Local Alignment Search, http://blast.ncbi.nlm.nih.gov/Blast.cgi?PROGRAM=blastp&PAGE_TYPE=BlastSearch&LINK_LOC=blasthome) tool [26] against the Protein Data Bank (PDB, http://www.rcsb.org/pdb/home/home.do) [27] in order to identify possible homologues proteins having tertiary structure resolved by experimental methods. The protein exhibiting the best BLAST/p e-value, alignment score and sequence coverage was used as template for building the 3D homology model of the *L. braziliensis* protein. The protein modeling was achieved using the Phyre2 server (http://www.sbg.bio.ic.ac.uk/phyre2/html/page.cgi?id=index) [28]. The predicted tertiary structures were compared with the predictions of secondary structures, and validated according to Ramachandran plot and other stereochemical parameters analyzed by the PROCHECK tool [29]. This program assesses the stereochemical quality of a given protein structure by analyzing the geometry of the residues in a given protein structure in comparison with stereochemical parameters derived from well-refined, high-resolution structures. The PROCHECK tool is available at the Swiss Model web-server (http://swissmodel.expasy.org/) [30]. Additionally, molecular modelling of the *L. braziliensis* RPA-1 sequence was obtained using the I-TASSER server, which has been developed to generate automated full-length tridimensional protein structural predictions [31-33]. The RPA-1 protein models were visualized with the program MacPyMOL Molecular Graphics System, Version 1.5.0.4.

## Results and discussion

### Structural features of the *L. braziliensis* RPA-1 and determination of its tridimensional structure by molecular modeling

**Figure 1:**
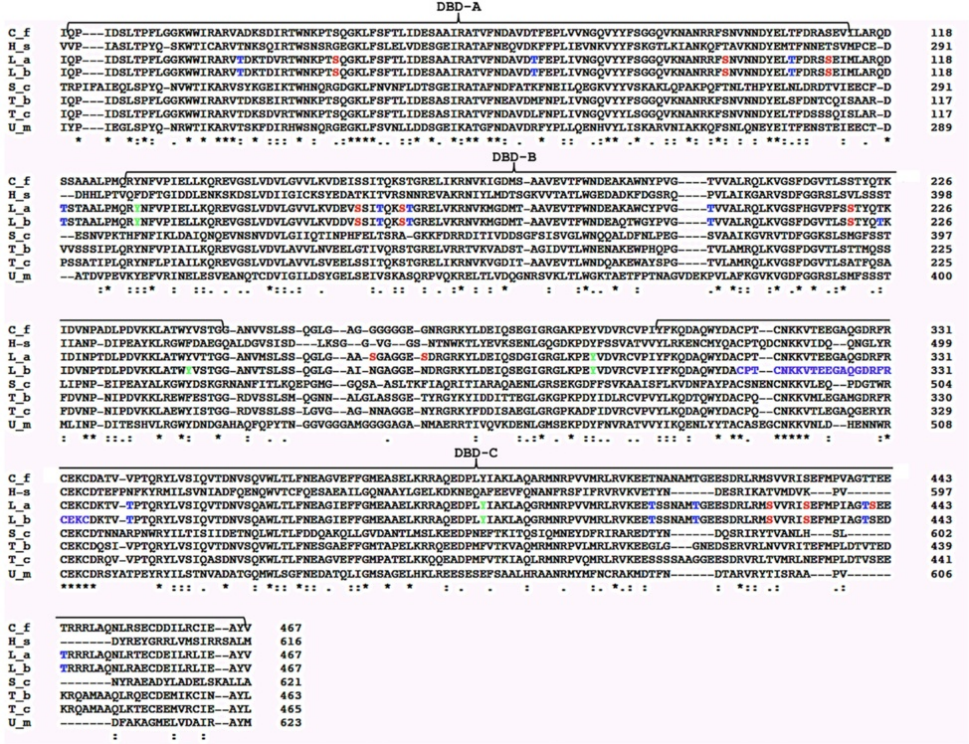
**Multiple alignment of RPA-1 sequences from trypanosomatids and evolutionarily divergent organisms.** Sequences were aligned with T-Coffee at the ExPASy. C_f, *C. fasciculata* (UniProtKB/Swiss-Prot: Q23696.1); H_s*, H. sapiens* (NCBI: NP_002936.1)*;* L_a, *L. amazonensis* (GenBank: AAR84278.1)*;* L_b, *L. braziliensis* (this work; GeneDB: LbrM.28.1990); S_c*, S. cerevisiae* (GenBank: AAC04960.1); T_b*, T. brucei* (GeneDB: Tb927.11.9130); T_c*, T. cruzi* (GeneDB: TcCLB.510901.60); U_m, *U. maydis* (GenBank: AAU05383.1)*.* Numbers on the right correspond to the amino acid position in the sequence. An asterisk means that amino acids are identical in all sequences shown in the alignment. A colon means that conserved substitutions are observed, and a period means that semi-conserved substitutions are observed. The location of the OB-fold domains (DBD) is indicated by brackets on the sequences. Letters in colors represent the potential phosphorylation sites in *L. amazonensis* and *L. braziliensis* RPA-1 proteins: red for serine, blue for threonine and green for tyrosine residues. The conserved zinc finger motif is indicated in magenta.

According to the data available at the GeneDB database, in the *L. braziliensis* genome exists a single-copy gene encoding RPA-1. The *LbRPA-1* gene is located at chromosome 28 (LbrM.28.1990), and it is defined by an ORF encoding for a protein of 467 amino acids. The genome of *Leishmania major* (MHOM/IL/81/Friedlin), *Leishmania infantum* (JPCM5), *Leishmania mexicana* (MHOM//GT/2001/U1103), *Leishmania donovani* (BPK282A1), *L. amazonensis* (Genbank accession number AAR84278.1) and *Leishmania tarentolae* (Parrot-TarII) contains also a single *RPA-1* gene, and in all species the predicted polypeptide has 467 amino acids. The alignment of *L. braziliensis* RPA-1 deduced amino acid sequence with homologous proteins from different organisms is shown in Figure 1. Among *Leishmania* species, sequence identity was higher than 95.9%; when compared with the homologues in other trypanosomatids (*Crithidia fasciculata, Trypanosoma brucei* and *Trypanosoma cruzi*) the sequence identity remained higher than 68.8%. Also significant sequence conservation (above 32.2% of sequence identity) was observed after comparison with RPA-1 proteins from evolutionarily distant organisms like *Homo sapiens, Saccharomyces cerevisiae* and *U. maydis.* As a remarkable feature, the RPA-1 protein in *Leishmania* and related trypanosomatids lacks an N-terminal domain (RPA-1 N), present in RPA-1 from the other organisms [13,17]. However, the other domains of RPA-1 (i.e., DBD-A, DBD-B and DBD-C) were reliably predicted in the *Leishmania* proteins (Figure 1). In addition, it was found that LbRPA-1, as described for other RPA-1 proteins [13,34,35], contains a C4-type zinc-finger motif, which, upon zinc coordination, stabilizes the tertiary structure of the RPA-1 C- terminal end and modulates DNA-binding [36]. The motif, C-X_2_-C-X_15_-C-X_2_-C, located at the 313 – 335 position in *L. braziliensis* sequence (Figure 1), is absolutely conserved in *C. fasciculata* [13], but slightly different to those found in human and yeast RPA-1 subunits (C-X_4_-C-X_13_-C-X_2_-C) [35]. Regarding the human sequence, the C-terminal region in *Leishmania* RPA-1 exhibits amino acid sequence differences, especially on the last 54 amino acids (Figure 1). Given the role of the C-terminal end for the trimerization of the RPA complex [36], and the fact that RPA protein has been considered as a target for cancer drug development [3,11], the differences at this region might be exploited for the discovery of molecules with pharmacological potential that perturb the complex formation of the RPA protein, following a similar approach to that used for the triosephosphate isomerase of *T. cruzi* [37].

RPA-1 undergoes phosphorylation, which is believed to play a role in modulating its affinity for DNA. In humans, the Chk1 protein, a serine/threonine kinase, phosphorylates with high efficiency the DBD-A and DBD-B domains (171–450 amino acids) [38]. As expected, it was noted that phosphorylation decreases the affinity of these two major DNA-binding domains [38]. Moreover, binding of RPA-1 to ssDNA blocks Chk1 phosphorylation, suggesting that Chk1 and ssDNA compete for binding to the RPA domains [38]. In this regard, the analysis of phosphorylation sites in *L. braziliensis* and *L. amazonensis* RPA-1 proteins showed the presence of at least 25 potential sites, sixteen of which are located on the DBD-A and DBD-B domains (Figure 1).

**Figure 2:**
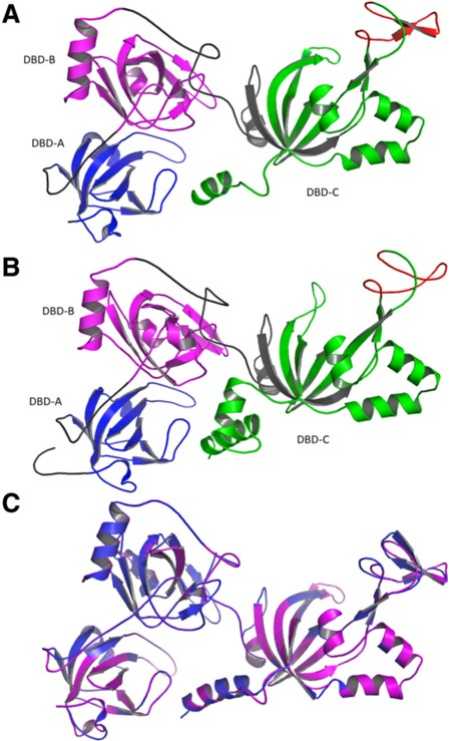
**Molecular modelling of** ***L. braziliensis*** **RPA-1 protein.** Tridimensional structures were obtained by Phyre2 (Panel **A**) and I-TASSER (Panel **B**) programs. OB-fold domains are indicated in colors as follows: DBD-A in blue, DBD-B in magenta and DBD-C in green. Panel **C**: structural alignment between the tridimensional structures of *L. braziliensis* RPA-1 and *U. maydis* RPA-1*.* The purpleblue structure corresponds to *U. maydis* RPA-1 protein, and the magenta one to *L. braziliensis* RPA-1 protein.

Molecular modeling of the *L. braziliensis* RPA-1 protein was carried out, firstly, using as template the *U. maydis* RPA-1 sequence (PDB identification number 4GOP). Both proteins share 34% of sequence identity (Figure 1). For crystallization purpose, the RPA-1 subunit of the fungus *U. maydis* was truncated to remove the N-terminal OB fold [19]. Nevertheless, as the *Leishmania* RPA-1 naturally lacks of this N-terminal domain (see above), most of LbRPA-1 sequence aligns with the resolved structure of *U. maydis* RPA-1. The truncated *U. maydis* RPA was crystallized bound to oligodeoxythymide ssDNA of either 62 nt or 32 nt, and the structures were refined at 2.8-A° and 3.1-A° resolution, respectively [19]. Interestingly, a tridimensional model covering from position 9 to 455 of LbRPA-1 was obtained using the Phyre2 program (Figure 2A). This model has been deposited in the Protein Model database (PMBD) with the ID PM0079361. The Ramachandran plot statistics showed that 81.4% of the residues in the modelled LbRPA-1 are in the most allowed regions, 15.9% in additional allowed regions, 2% in generously allowed regions and only 0.8% in disallowed regions. These data indicate that the *Leishmania* modelled protein has a quality similar to that determined for the *U. maydis* X-ray resolved structure. Thus, the Ramachandran plot data indicated that the build model is appropriate and suitable for further analyses. Additionally, the LbRPA-1 sequence was submitted to the iterative threading assembly refinement (I-TASSER) server, which carry out a fully automated protein prediction based on iterative structural assemblies with tridimensional models of known proteins. The more probable structure generated by this method (Figure 2B) resulted to be very similar to that generated by Phyre2 program (Figure 2A). Unsurprisingly, the I-TASSER server also found the *U. maydis* RPA-1 as the more adequate model for modelling of LbRPA-1. In panel C of Figure 2, it is shown the structural alignment of both protein models (LbRPA-1 and UmRPA-1) that was generated using the MacPyMOL program. A root mean-square deviation (RMSD) value of 0.109 Å was obtained; this value suggests that, in spite of the genetic distance between these two types of organisms, the overall structure of both RPA-1 is maintained.

**Figure 3:**
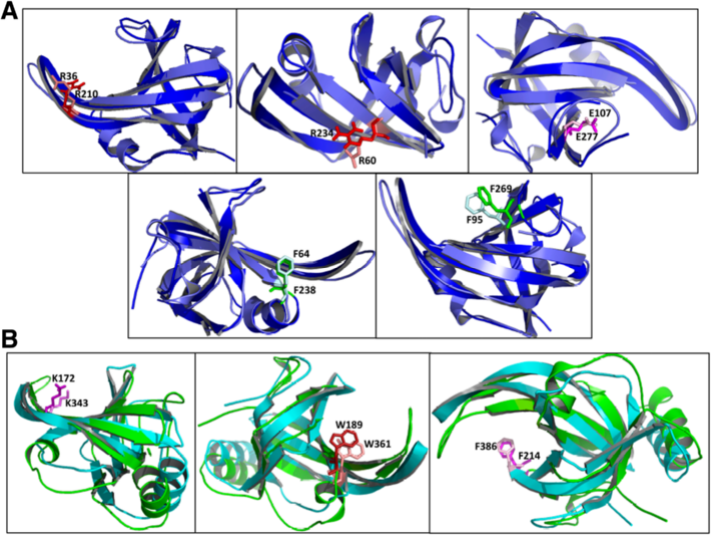
**Superposition of structures and relevant amino acids between LbRPA-1 and hRPA-1 3D models in the DBD-A (panels A) and DBD-B (panels B) domains.** In panels **A**, light coloured structures correspond to LbRPA-1 and dark coloured ones to hRPA-1. In panels **B**, light colors were used for hRPA-1 and dark colors for LbRPA-1.

In the human RPA-1 (hRPA-1) several amino acids have been identified as crucial for DNA binding [7], we analyze if they were also present in equivalent structural positions in the LbRPA-1. Remarkably, R210, R234, F238, F269 and E277 residues in the DBD-A domain of hRPA-1 were found conserved in the DBD-A domain of LbRPA-1; they corresponds to the residues R36, R60, F64, F95 and E103, respectively (Figure 3A). Similarly, the K172, W189 and F214 amino acids of the LbRPA-1 protein would be the equivalent to residues K343, W361 and F386 described as crucial for DNA-binding in the DBD-B domain of hRPA-1 (Figure 3B). In all, these findings pointed to a functional conservation of the DNA-binding capacity between hRPA-1 and the *L. braziliensis* LbRPA-1 protein.

### Expression of *L. braziliensis* RPA-1 as recombinant protein and analysis of its affinity to ssDNA

**Figure 4:**
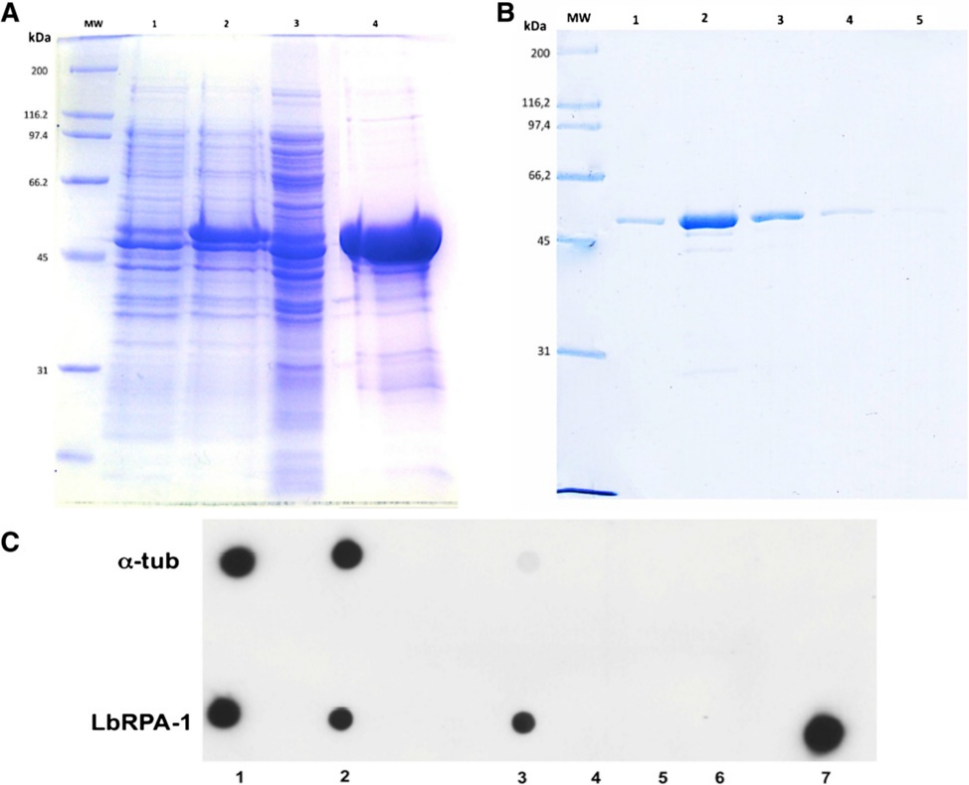
**Expression, purification and functional analysis of rLbRPA-1.** Panel **A**. Analysis of the LbRPA-1 expression in *E. coli* – M15 cells. Lanes: 1, total cell extract before induction; 2, total cell extract after inducing with IPTG; 3, soluble fraction of the cell extract; 4, insoluble fraction of the cell extract; MW, molecular weight markers. Panel **B**, Analysis by Coomassie blue staining of the purified rLbRAP-1. Lanes 1 to 4 correspond to sequential fractions of eluted protein after affinity chromatography and in-column refolding procedure. Panel **C**, functional analysis of the rLbRPA-1 protein. *L. braziliensis* recombinant α-tubulin (α-tub) or LbRPA-1 were incubated with a digoxigenine labelled oligonucleotide (DIG-O) following the protocol described in Materials and Methods**.** Dots: 1, amount of DIG-O used in the binding assay; 2, unbound DIG-O; 3–6, DIG-O washed out in sequential washed; 7, eluted protein-DIG-O complexes.

For expression of recombinant *L. braziliensis* RPA-1, oligonucleotides were designed from the GeneDB entry LbrM.28.1990 (see Methods for further details), and the coding region was cloned into the *E. coli* pQE30 expression vector. The expression of the protein was observed after IPTG addition to the culture medium (Figure 4A). However, subcellular fractionation evidenced that most of the protein was forming insoluble polypeptide aggregates, i.e. inclusion bodies. A similar finding was observed by Lira and co-workers when expressing the *L. amazonensis* RPA-1 in *E. coli* [25]. In order to solubilize the protein, these authors developed a purification protocol that allowed to recover the protein in a highly pure and active form. Therefore, we followed that procedure with minimal modifications (see Methods), and finally it was possible to obtain the *L. braziliensis* rRPA-1 in a soluble form (Figure 4B).

After purification of the rLbRPA-1, we analyzed whether the protein maintained its cognate capacity of binding to ssDNA. For this purpose, an oligodeoxyribonucleotide, containing a digoxigenin molecule at its 5′-end (DIG-O), was synthetized and used in binding assays with the recombinant protein. The ability of the rLbRPA-1 to bind this DIG-O was tested by pull-down assays based on the affinity of the His-tag to Ni^2+^-beads. As shown in Figure 4C, rLbRPA-1 showed a clear affinity for ssDNA, suggesting that the protein was refolded *in vitro* correctly. As a negative control, binding assays using recombinant *L. braziliensis* α-tubulin were performed in parallel.

**Figure 5:**
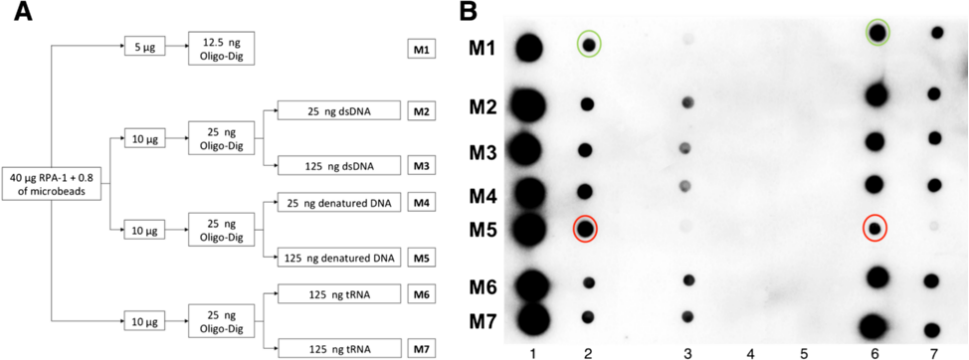
**Qualitative analysis of the affinity of rLbRPA-1 for ssDNA.** Panel **A**, scheme showing the experimental design. Panel **B**, follow up of the digoxigenin-labelled oligonucleotide (Oligo-Dig) in the different steps of the pull down assay. For each experiment, the analyzed samples were: 1, total amount of oligo-DIG used in the assay (12.5 ng); 2, unbound oligo-DIG after incubation with rLbRAP-1 containing beads; 3–5, Oligo-DIG presents in sequential washes; 6 and 7, 1/4 and 1/16 dilutions, respectively, of the eluted rLbRPA-1-oligo-DIG complexes. Green circles in samples two and six from mixture 1 (M1) indicate, respectively, unbound (2) and 1/4 of the eluted oligonucleotide (6), as signal intensity control, whilst red circles mark equivalent samples of the mixture 5 (M5) in which the oligo-DIG binding was compited by ssDNA.

To further characterize the substrate specificity of rLbRPA-1, competition assays using dsDNA, ssDNA or tRNA were carried out (Figure 5). After binding of rLbRPA-1 to the DIG-O, only a five molar excess of ssDNA was able to significant displace the bound DIG-O. No competition was observed when dsDNA was used in excess. Also, no displacement of the DIG-O was attained after incubation with yeast tRNA (Figure 5). In summary, from these experiments, it must be concluded that rLbRPA-1, as described for homologous proteins in other organisms, is essentially a ssDNA binding protein.

### *L. braziliensis* RPA-1 also binds RNA

**Figure 6:**
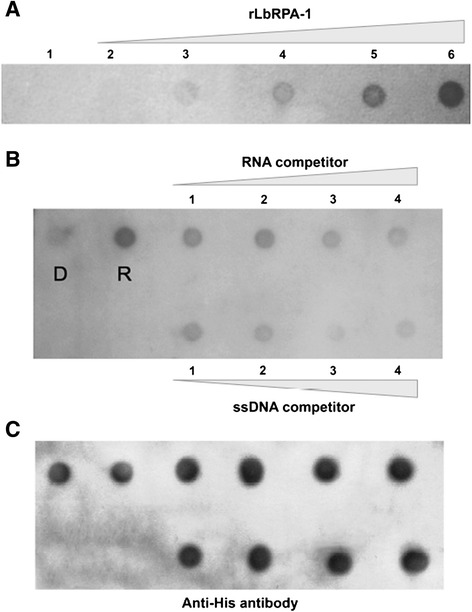
**Analysis of the RNA-binding capacity of the rLbRPA-1 protein and its relative affinity for RNA and ssDNA.** (Panel **A**) Two hundred ng of digoxigenin labeled 3′ HSP70 UTR-II were incubated with beads containing different amounts of rLbRPA-1: 0 (dot 1), 0.05 pM (2), 0.25 pM (3), 1.25 pM (4), 6.25 pM (5) and 31.25 pM (6). After whasing out of the unbound RNA, eluted complexes were deposited on the membrane and the remaining bound RNA was monitored by an anti-digoxigenin antibody. (Panel **B**) One pM of either denatured (dot D) or renatured rLbRPA-1 (dot R) was incubated with the digoxigenin-labeled RNA oligonucleotide (100 fM). Additionally, the binding of the Dig-RNA oligonucleotide to the renatured rLbRPA-1 was competited with increasing amounts of either the non-labelled RNA oligonucleotide (upper array) or a non-labelled DNA oligonucleotide (lower array) containing the equivalent sequence: 25 fM (dot 1), 50 fM (2), 100 fM (3) and 200 fM (4). After whasing out the unbound oligonucleotides, the complexes were eluted and put on the membrane, which was revealed by an anti-digoxigenine antibody. (Panel **C**) The membrane of panel B was incubated with an anti-his-tag antibody in order to monitor that equivalent amounts of rLbRPA-1 was present in the different samples.

Although the rLbRPA-1 showed to have a clear affinity for ssDNA, we addressed experimentally whether the protein can also interact with RNA. It should be noted that this protein was initially identified by its binding to the untranslated regions of the *HSP70* mRNAs [18]. On the other hand, Cano and co-workers described that *L. amazonensis* RPA-1 was able to interact with an RNA oligonucleotide containing the *Tetrahymena* telomeric sequence [15]. Moreover, there are many proteins of the *T. brucei* editosome, involved in RNA editing, that contains the OB-fold domain [39]. In order to study an rLbRPA-1/RNA interaction, we designed non-competitive and competitive pull-down assays. For this purpose, after purifying the recombinant LbRPA-1, it was bound to Ni^2+^-beads and incubated with digoxigenin-labeled RNA containing the 3′UTR of *L. braziliensis HSP70-II* gene. Interestingly, it was observed that rLbRPA-1 interacts with RNA and the amount of bound RNA increased in parallel with the amount of protein present in the sample (Figure 6A). These results indicated that rLbRPA-1 has also RNA-binding activity.

Once the RNA binding activity of rLbRPA-1 was demonstrated, we considered of interest to further evaluate its relative affinity for DNA and RNA. For this purpose, a digoxigenin-labelled RNA oligonucleotide (derived from the 3′ *HSP70-II* UTR RNA sequence; 5′ 5DigN-AUGUCUUUUA UUUUUUUGUG UGUGUUUUAU AUUUUUCUCC UUUCGUACUA A 3′) was incubated with the renatured rLbRPA-1 and its binding analyze also by pull-down assays (Figure 6B). In this assay, we included an assay in which the rLbRPA-1 was maintained in a denatured conformation by adding 8 M urea to the binding buffer. This control served to exclude that the oligonucleotide binding was non-specific (Figure 6B, point D). Additionally, competition assays using either the non-labelled RNA oligonucleotide or a DNA oligonucleotide containing the equivalent sequence (5′ ATGTCTTTTA TTTTTTTGTG TGTGTTTTAT ATTTTTCTCC TTTCGTACTA A 3′) were carried out. The results showed that the competition was more effective in the presence of the DNA oligonucleotide competitor than with the RNA one. These findings confirmed the higher affinity of rLbRPA-1 for ss-DNA than for RNA, at least when this particular sequence, present in the 3′ *HSP70-II* mRNA molecule, is used as substrate. Nevertheless, for future works, it should be analyzed the relative affinity for DNA and/or RNA of the native LbRPA-1, considering that heterologous expression of rLbRPA-1 (as for any other protein) in *E. coli* could have introduced conformational alterations or modifications that may affect in some extent the affinity for its targets.

## Conclusions

The rLbRPA-1 protein, purified in denaturing conditions and refolded *in vitro*, was able to bind ssDNA molecules. This result confirmed, on one hand, that the purified protein is functional and, on the other hand, allowed us to confirm that LbRPA-1 is a ssDNA binding protein as predicted. In addition, we have shown that this protein is able to interact with RNA molecules, which may be suggesting a role of this protein in the mRNA *HSP70* expression, in particular, and in RNA metabolism, in general. Likewise, the detailed structural analysis and the molecular modelling carried out on the *Leishmania* RPA-1 protein have shown a remarkable conservation with the homologues from evolutionarily distant organisms. Nevertheless, the comparison with the human RPA-1 protein also served to locate differences, such as the absence of the N-terminal RPA70N domain and the existence of a highly divergent sequence at the C- terminal region. These differences could be exploited for developing molecules with antileishmanial properties.
